# Dietary patterns during pregnancy and maternal and birth outcomes in women with type 1 diabetes: the Environmental Determinants of Islet Autoimmunity (ENDIA) study

**DOI:** 10.1007/s00125-024-06259-5

**Published:** 2024-09-02

**Authors:** Rebecca L. Thomson, James D. Brown, Helena Oakey, Kirsten Palmer, Pat Ashwood, Megan A. S. Penno, Kelly J. McGorm, Rachel Battersby, Peter G. Colman, Maria E. Craig, Elizabeth A. Davis, Tony Huynh, Leonard C. Harrison, Aveni Haynes, Richard O. Sinnott, Peter J. Vuillermin, John M. Wentworth, Georgia Soldatos, Jennifer J. Couper, on behalf of the ENDIA Study Group

**Affiliations:** 1 Adelaide Medical School, Faculty of Health and Medical Sciences and Robinson Research Institute, University of Adelaide, Adelaide, SA Australia; 2 Monash Women’s, Monash Health, Clayton, VIC Australia; 3 Department of Obstetrics and Gynaecology, Monash University, Clayton, VIC Australia; 4 Department of Nutrition & Food Services, Women’s and Children’s Hospital, North Adelaide, SA Australia; 5 Department of Diabetes and Endocrinology, Royal Melbourne Hospital, Parkville, VIC Australia; 6 School of Women’s and Children’s Health, Faculty of Medicine, University of New South Wales, Randwick, NSW Australia; 7 Institute of Endocrinology and Diabetes, The Children’s Hospital at Westmead, Westmead, NSW Australia; 8 Telethon Kids Institute, Centre for Child Health Research, The University of Western Australia, Nedlands, WA Australia; 9 Endocrinology and Diabetes, Perth Children’s Hospital, Nedlands, WA Australia; 10 Department of Endocrinology and Diabetes, Queensland Children’s Hospital, South Brisbane, QLD Australia; 11 Children’s Health Research Centre, The University of Queensland, South Brisbane, QLD Australia; 12 Walter and Eliza Hall Institute of Medical Research, Parkville, VIC Australia; 13 Department of Medical Biology, University of Melbourne, Melbourne, VIC Australia; 14 Melbourne eResearch Group, School of Computing and Information Services, University of Melbourne, Melbourne, VIC Australia; 15 Faculty of Health, School of Medicine, Deakin University, Geelong, VIC Australia; 16 Child Health Research Unit, Barwon Health, Geelong, VIC Australia; 17 School of Public Health and Preventive Medicine, Faculty of Medicine, Nursing and Health Sciences, Monash University, Clayton, VIC Australia; 18 School of Clinical Sciences, Faculty of Medicine, Nursing and Health Sciences, Monash University, Clayton, VIC Australia; 19 Diabetes and Vascular Medicine Unit, Monash Health, Clayton, VIC Australia; 20 Endocrinology and Diabetes Department, Women’s and Children’s Hospital, North Adelaide, SA Australia

**Keywords:** Birth outcomes, Dietary patterns, Pre-eclampsia, Pregestational diabetes, Pregnancy complications, Type 1 diabetes

## Abstract

**Aims/hypothesis:**

Dietary patterns characterised by high intakes of vegetables may lower the risk of pre-eclampsia and premature birth in the general population. The effect of dietary patterns in women with type 1 diabetes, who have an increased risk of complications in pregnancy, is not known. The aim of this study was to investigate the relationship between dietary patterns and physical activity during pregnancy and maternal complications and birth outcomes in women with type 1 diabetes. We also compared dietary patterns in women with and without type 1 diabetes.

**Methods:**

Diet was assessed in the third trimester using a validated food frequency questionnaire in participants followed prospectively in the multi-centre Environmental Determinants of Islet Autoimmunity (ENDIA) study. Dietary patterns were characterised by principal component analysis. The Pregnancy Physical Activity Questionnaire was completed in each trimester. Data for maternal and birth outcomes were collected prospectively.

**Results:**

Questionnaires were completed by 973 participants during 1124 pregnancies. Women with type 1 diabetes (*n*=615 pregnancies with dietary data) were more likely to have a ‘fresh food’ dietary pattern than women without type 1 diabetes (OR 1.19, 95% CI 1.07, 1.31; *p*=0.001). In women with type 1 diabetes, an increase equivalent to a change from quartile 1 to 3 in ‘fresh food’ dietary pattern score was associated with a lower risk of pre-eclampsia (OR 0.37, 95% CI 0.17, 0.78; *p*=0.01) and premature birth (OR 0.35, 95% CI 0.20, 0.62, *p*<0.001). These associations were mediated in part by BMI and HbA_1c_. The ‘processed food’ dietary pattern was associated with an increased birthweight (β coefficient 56.8 g, 95% CI 2.8, 110.8; *p*=0.04). Physical activity did not relate to outcomes.

**Conclusions/interpretation:**

A dietary pattern higher in fresh foods during pregnancy was associated with sizeable reductions in risk of pre-eclampsia and premature birth in women with type 1 diabetes.

**Graphical Abstract:**

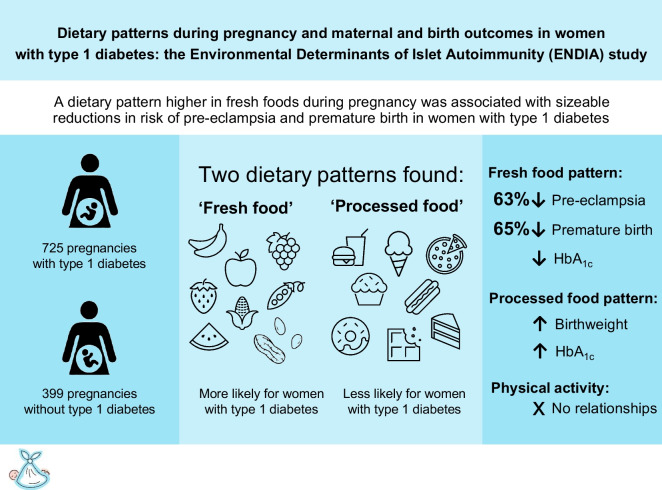

**Supplementary Information:**

The online version of this article (10.1007/s00125-024-06259-5) contains peer-reviewed but unedited supplementary material.


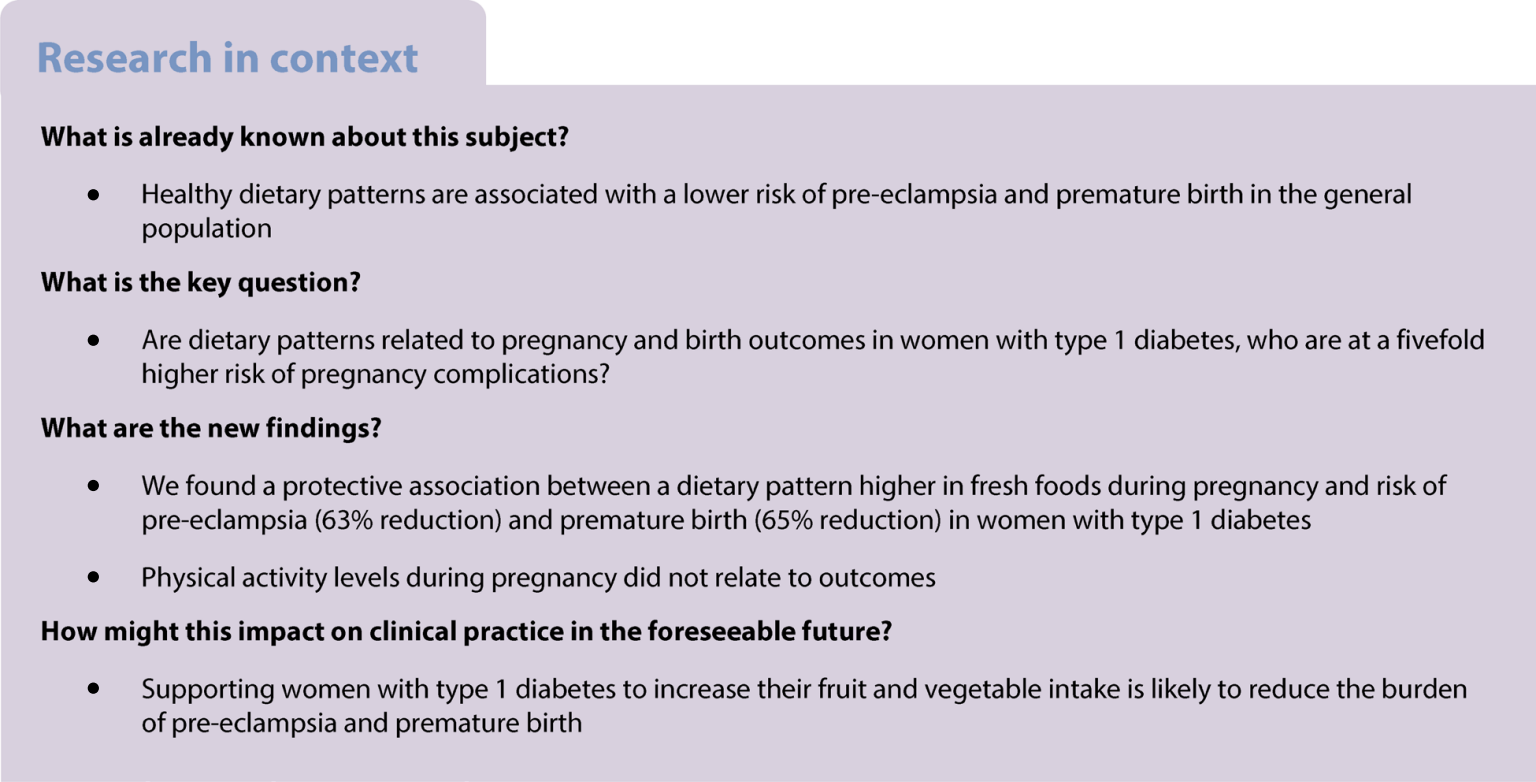


## Introduction

Women with type 1 diabetes have a substantially increased risk of adverse maternal and perinatal outcomes, including pre-eclampsia, premature birth and infants born large for gestational age [1, 2]. Healthier dietary patterns during pregnancy have been associated with more favourable pregnancy and birth outcomes in the general population [3–5]. Analysis of dietary patterns has the advantage of examining the effect of overall diet, rather than being restricted to individual nutrients or foods. By providing a broader picture of food and nutrient consumption, dietary patterns may be more predictive of disease risk [6]. In the general population, dietary patterns during pregnancy characterised by high intakes of vegetables, plant foods and vegetable oils, or a high adherence to a Mediterranean-style dietary pattern pre-pregnancy, are associated with a moderately lower risk of developing hypertensive disorders of pregnancy (HDP), including pre-eclampsia [3, 4]*.* Dietary patterns characterised by high intakes of vegetables, fruits, wholegrains, low-fat dairy and lean protein foods are associated with a lower risk of preterm birth [5] and those characterised by high intakes of refined grains, processed meat and foods high in saturated fat or sugar with lower birthweight [5]. Increased physical activity and reduced sedentary behaviour during pregnancy have also been associated with moderately reduced risk of pre-eclampsia and gestational hypertension in the general population [7, 8].

Attention to nutrition is essential for women with type 1 diabetes during pregnancy, both for optimal glycaemic control and to meet increased nutritional requirements. However, to date no studies have investigated the impact of dietary intake during pregnancy on maternal and birth outcomes in women with type 1 diabetes.

The primary aim of this study was to investigate the relationship between dietary patterns and physical activity during pregnancy with maternal complications and birth outcomes in a large cohort of women with type 1 diabetes followed prospectively in the Environmental Determinants of Islet Autoimmunity (ENDIA) study. We also aimed to compare their dietary patterns with women without type 1 diabetes who also participated in ENDIA.

## Methods

### Study design and participants

Data were collected prospectively as part of the ENDIA study, a national Australian longitudinal prospective pregnancy/birth cohort study with the overall aim to determine the early-life exposures that drive the development of type 1 diabetes [9]. In this study, all women were investigated according to the ENDIA protocol at 3 month intervals during pregnancy from the time of recruitment until birth, and their children in the neonatal period. Investigation included clinical measurements, clinical outcomes and questionnaires assessing nutrition, exercise and lifestyle.

ENDIA recruited 1488 pregnant women or those with a child less than 6 months of age between February 2013 and November 2019, where the child had a first-degree relative with type 1 diabetes. Women were excluded from the study if they had an inadequate understanding of English to provide consent and responses to questionnaires. This analysis included all women who completed at least one diet or physical activity questionnaire during pregnancy; women with twin or triplet pregnancies were excluded.

The ENDIA study was reviewed and approved by the study’s lead Human Research Ethics Committee at the Women’s and Children’s Health Network under the National Mutual Acceptance Scheme (current approval no. 2020/HRE01400) and at all participating study sites. Conduct in Western Australia was approved by the Women and Newborn Health Service Ethics Committee (ref. no. RGS0000002639). ENDIA is registered on the Australian New Zealand Clinical Trials Registry (ACTRN12613000794707). All women provided written informed consent and were free to withdraw from the study at any time.

### Demographic and clinical data

Pre-pregnancy weight and maternal demographics were self-reported by participants at their first appointment. Pre-pregnancy BMI (kg/m^2^) was calculated using pre-pregnancy reported weight and height measured at the first visit. Maternal weight was measured at each visit and additional weight measurements collected at routine clinic visits were obtained from medical records. If pre-pregnancy weight was missing, the earliest weight available in the first trimester was used to estimate pre-pregnancy BMI. Gestational weight gain was calculated as the difference between the last weight measured during the third trimester of pregnancy and the pre-pregnancy weight or first weight recorded during pregnancy. Pregnancy data (including parity, HbA_1c_, medications and medical complications) and birth outcomes (gestational age at birth, birthweight and neonatal hypoglycaemia) were obtained from hospital medical records. Socioeconomic status was calculated using postcode at enrolment using the Socio-Economic Indexes for Areas Index of Relative Socio-Economic Advantage and Disadvantage (SEIFA IRSAD). This index is derived from national Census variables related to both advantage and disadvantage, for example household income and level of education [10]. Remoteness was classified using the Modified Monash Model [11], which defines a location according to geographical remoteness, as defined by the Australian Bureau of Statistics, and town size.

HDP were verified using medical record review according to International Society for the Study of Hypertension in Pregnancy classification [12] and included gestational hypertension (new onset of hypertension after 20 weeks’ gestation), pre-eclampsia (hypertension occurring for the first time after 20 weeks, associated with proteinuria and/or organ involvement), superimposed pre-eclampsia (pre-eclampsia superimposed on chronic hypertension), eclampsia and HELLP (haemolysis, elevated liver enzymes and low platelets) syndrome. Prematurity was defined as birth before 37 weeks’ gestation. Neonatal hypoglycaemia was defined as blood glucose level in the newborn of less than 2.6 mmol/l within the first 72 h post birth.

HbA_1c_ was measured using either point-of-care or laboratory testing methods, commonly a Vantage analyser (Siemens Diagnostics, Camberley, UK) or a Variant analyser (Bio-Rad Laboratories, Hercules, CA, USA). All medical laboratories were accredited by the National Association of Testing Authorities, Australia, against the international standard ISO 15189 Medical laboratories, which mandates that all analytes in a laboratory’s test menu be subject to the Royal College of Pathologists of Australasia Quality Assurance Programs [13]. The first HbA_1c_ measurement available during pregnancy (usually conception or first trimester) was used and analysis controlled for gestational week of measurement.

### Dietary and lifestyle measures

Diet was assessed using the Dietary Questionnaire for Epidemiological Studies version 2 (DQESv2), a self-administered 74-item food frequency questionnaire [14] validated in women of child-bearing age (16–48 years) [15]. Participants completed the questionnaire during their third trimester of pregnancy and were asked to assess their diet since the start of pregnancy. The DQESv2 provided daily intakes (in grams) of specific foods and beverages. The 101 individual food items were combined into 19 food item categories based on nutrient content and culinary usage [16] (electronic supplementary material [ESM] Table 1) for the analysis of dietary patterns. Consumption of each food item was converted into daily servings by adjusting the intake for serving size as described in the Australian Dietary Guidelines [17]. The total number of servings per day were calculated by summing the numbers of servings consumed per day for all food items in each of the five food groups of the Australian Dietary Guidelines.

Physical activity was measured during each trimester using the Pregnancy Physical Activity Questionnaire (PPAQ) [18], a validated self-report questionnaire that measures the time spent participating in 32 activities grouped into different types of activity (i.e. sedentary, light, moderate and vigorous activity). Participants could add two physical activities not listed in the questionnaire, where the intensities were individually estimated using the Compendium of Physical Activities [19]. The duration of time spent in each activity was multiplied by its intensity (i.e. metabolic equivalent of task [MET]) and summed to calculate the mean weekly energy expenditure, expressed as MET-hours/week.

### Statistical analysis

To account for the potential correlation between data from the same participant during different pregnancies (i.e. a participant included in the study more than once), a random intercept for each participant was included in each model. Analyses were restricted to participants with complete data as missing data were minimal for outcomes and confounders used in analyses (as reported in tables). R statistical software version 4.3.1 [20] and a significance level of 5% was used for all analyses. Results for adjusted models are reported unless otherwise specified.

### Dietary patterns for pregnancies with and without type 1 diabetes

Dietary patterns were derived using principal component analysis (PCA) on dietary data based on 19 food item categories from all women with and without type 1 diabetes. The resulting principal components, derived in decreasing order of importance, were a linear combination of the food items. The number of dietary patterns (principal components) identified was based on eigenvalues >1.5 and on identification of a break point in the scree plot [21]. Food item categories with a factor loading of ±0.30 or more were considered important contributors of each dietary pattern [22]. Scores for each principal component were obtained by summing up observed intakes of the component food items weighted by the factor loading and indicate the extent to which the participant’s diet conformed to the respective dietary pattern. A logistic regression mixed model was used to compare dietary patterns in women with and without type 1 diabetes using the principal component scores from each dietary pattern. Potential confounders (age, pre-pregnancy BMI and parity) were included in the model.

### Association between dietary patterns and physical activity, and maternal complications and birth outcomes in pregnancies with type 1 diabetes

Analysis of associations between dietary patterns and outcomes were planned only for the women with type 1 diabetes, as maternal complications and adverse birth outcomes are about fivefold more prevalent in women with type 1 diabetes in Australia. Maternal outcomes were HDP (categorised as gestational hypertension or pre-eclampsia/eclampsia/HELLP), and birth outcomes were prematurity, gestational age at birth, birthweight and neonatal hypoglycaemia. Exposures were dietary patterns (participant principal components scores) and physical activity (mean MET-hours/week for total activity, sedentary activity, and moderate and vigorous activity). Models that investigated associations with physical activity included nested random intercept terms, visits within pregnancies within the same participant (to account for the fact that each mother could have completed up to three questionnaires for each pregnancy), and were adjusted for the gestational age when the questionnaire was completed.

Prematurity (<37 weeks’ gestation) and neonatal hypoglycaemia (blood glucose level <2.6 mmol/l) were fitted in separate mixed logistic regression models. A separate mixed multinomial logistic regression model was used for the HDP categories. For continuous outcomes—HbA_1c_, gestational age at birth and birthweight—separate linear mixed models were fitted. Potential confounders (maternal age, parity and SEIFA IRSAD percentile) were adjusted for in all models. Birthweight was also adjusted for gestation at birth. To make the results more interpretable, participant scores identified from PCA were rescaled such that a one-unit change in principal component score represented the IQR from 25th percentile to 75th percentile. The mean intake of food group servings was calculated from participants in each quartile of principal component scores for the ‘fresh food’ dietary pattern. The difference in intake between the highest and lowest quartile was calculated to correspond to a one-unit change of the rescaled scores.

### Mediation analyses

Potential mediators of the association between dietary pattern and pre-eclampsia and premature birth were HbA_1c_ and BMI. Model-based causal mediation analysis with quasi-Bayesian Monte Carlo simulation (10,000 simulation) was performed [23] using the ‘mediation’ R package. While pre-pregnancy BMI is not strictly a mediator temporally between diet and pre-eclampsia (i.e. it was measured before the time covered by the diet questionnaire), it is an available proxy for early pregnancy BMI before weight increases. Therefore, the influence of pre-pregnancy BMI was also investigated as a mediator between diet and pre-eclampsia using model-based causal mediation analysis.

### Sensitivity analyses

Sensitivity analyses were conducted excluding participants from the PCA if they reported an unrealistic energy intake (energy <4500 kJ/day or >20,000 kJ/day, *n*=109, including 82 with type 1 diabetes) [24] and if they had gestational diabetes (*n*=51) or type 2 diabetes (*n*=1). All models were then refitted with the resulting principal components scores. Separate sensitivity analyses were also conducted for the outcomes of pre-eclampsia and prematurity which excluded women with a parity greater than 0 (i.e. included only nulliparous women) as the risk of pre-eclampsia and premature birth are substantially influenced by parity and complications in a prior pregnancy.

## Results

### Participants

**Fig. 1:**
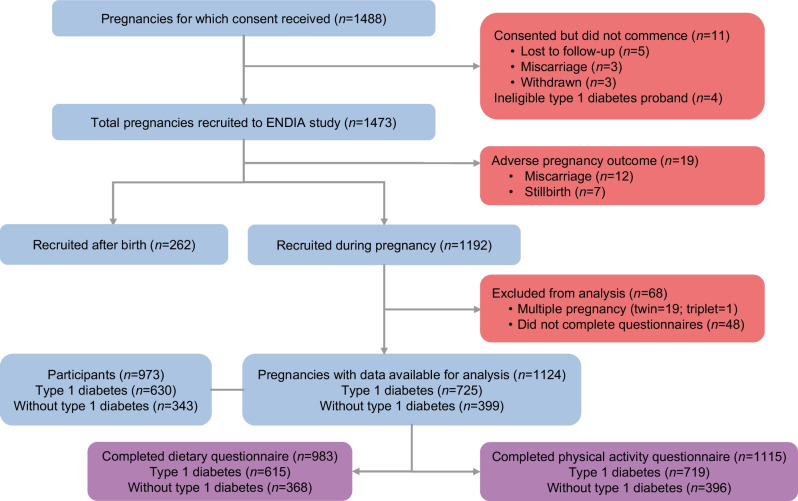
STROBE (Strengthening the reporting of observational studies in epidemiology) diagram of participants included in the analysis

This analysis included 973 women who had 1124 pregnancies (725 pregnancies with type 1 diabetes and 399 pregnancies without type 1 diabetes) (Fig. 1). The 1124 pregnancies were representative of the full ENDIA cohort (1453 pregnancies and 1214 unique participants) in terms of maternal age, BMI, parity, socioeconomic demographics, and including the relative proportion of participants with and without type 1 diabetes [25]. Clinical characteristics and pregnancy and birth outcomes for participants included in this analysis are outlined in Table 1. Premature birth was mainly iatrogenic via induction of labour (22%) or Caesarean birth without labour (58%). Spontaneous premature labour occurred for the remaining 20% of participants with premature birth. A substantially higher proportion of women with type 1 diabetes had maternal complications and adverse birth outcomes (Table 1).

**Table 1:** Demographic characteristics, maternal complications and birth outcomes of pregnancies in women with and without type 1 diabetes

Variable	Pregnancies with type 1 diabetes (*n*=725)	Pregnancies without type 1 diabetes (*n*=399)
Age at delivery (years)	31.7 (4.6)	33.1 (4.3)
Born in Australia, *n* (%)	596 (82)	315 (79)
Missing	8 (1)	5 (1)
IRSAD, *n* (%)		
Quintile 1 (most disadvantaged)	68 (9)	44 (11)
Quintile 2	95 (13)	41 (10)
Quintile 3	136 (19)	74 (19)
Quintile 4	198 (27)	103 (26)
Quintile 5 (least disadvantaged)	228 (31)	137 (34)
Remoteness, *n* (%)		
Metropolitan area	600 (83)	345 (86)
Regional centre and rural town	114 (16)	53 (13)
Remote and very remote community	11 (2)	1 (<1)
Education, *n* (%)		
Tertiary	572 (79)	334 (84)
Secondary or less	146 (20)	63 (16)
Not given	7 (1)	2 (1)
Parity, *n* (%)		
0	362 (50)	148 (37)
1	264 (36)	144 (36)
2+	99 (14)	107 (27)
Pre-pregnancy BMI (kg/m^2^)	24.9 (21.2–28.3)	25.5 (22.8–30.0)
Obesity (BMI >30 kg/m^2^), *n* (%)	183 (25)	65 (16)
Missing	5 (1)	3 (1)
Gestational weight gain (kg)	13.0 (9.9–16.9)	13.0 (10.0–15.3)
Missing, *n* (%)	90 (12)	44 (11)
Age at type 1 diabetes diagnosis (years)	13.4 (8.5–21.1)	–
Missing, *n* (%)	27 (4)	–
HbA_1c_ (mmol/mol)	51 (44–58)	–
HbA_1c_ (%)	6.8 (6.2–7.5)	–
Missing, *n* (%)	14 (2)	–
Any smoking during pregnancy, *n* (%)	56 (8)	20 (5)
Missing	8 (1)	9 (2)
Any alcohol during pregnancy, *n* (%)	132 (18)	99 (25)
Missing	110 (15)	31 (8)
Supplements containing folic acid, *n* (%)	699 (96)	374 (94)
Supplements containing iron, *n* (%)	688 (95)	374 (94)
Physical activity during pregnancy (MET-hours/week)		
Total physical activity	276 (116)	275 (116)
Sedentary activity	71 (30)	70 (32)
Moderate and vigorous activity	100 (80)	96 (80)
Missing, *n* (%)	6 (1)	3 (1)
HDP, *n* (%)	194 (27)	30 (8)
Gestational hypertension	71 (10)	22 (6)
Pre-eclampsia	95 (13)	7 (2)
Superimposed pre-eclampsia	21 (3)	0 (0)
Eclampsia	0 (0)	0 (0)
HELLP syndrome	7 (1)	1 (<1)
Missing	6 (1)	4 (1)
Pre-existing hypertension, *n* (%)	46 (6)	7 (2)
Antihypertensive use pre-pregnancy, *n* (%)	36 (5)	6 (2)
History of previous HDP, *n* (%)	52 (7)	19 (5)
Mode of delivery, *n* (%)		
Caesarean	519 (72)	139 (35)
Vaginal	203 (28)	259 (65)
Missing	3 (<1)	1 (<1)
Onset of labour, *n* (%)		
Spontaneous	84 (12)	176 (44)
Induction	286 (39)	132 (33)
No labour	352 (49)	90 (23)
Missing	3 (<1)	1 (<1)
Gestational age (weeks)	37.3 (36.1–38.0)	39.1 (38.3–40.1)
Premature (<37 weeks), *n* (%)	284 (39)	20 (5)
Term (37–41 weeks), *n* (%)	438 (60)	377 (94)
Post-term (42+ weeks), *n* (%)	0 (0)	1 (<1)
Missing, *n* (%)	3 (<1)	1 (<1)
Birthweight (g)	3645 (3220–4010)	3503 (3165–3773)
Small for gestational age^a^, *n* (%)	9 (1)	18 (5)
Large for gestational age^b^, *n* (%)	462 (64)	62 (16)
Missing, *n* (%)	4 (1)	1 (<1)
Neonatal hypoglycaemia, *n* (%)	518 (71)	59 (15)
Missing/not measured	3 (<1)	176 (44)

Values are presented as mean (SD), median (IQR; quartile 1–quartile 3) or *n* (%)

If missing data are not included, there were no missing values for that variable

^a^Small for gestational age defined as ≤10th centile from population-based Australian birthweight centile charts [43]

^b^Large for gestational age defined as ≥90th centile from population-based Australian birthweight centile charts [43]

^c^Recommended daily number of servings from each food group during pregnancy reported in parentheses [17]

The DQESv2 was completed during 983 pregnancies with and without type 1 diabetes at a median gestational age of 33.8 (IQR 32.1–35.9) weeks. The PPAQ was completed during 406 pregnancies in early pregnancy at a median gestation of 12.5 (IQR 10.1–14.7) weeks, 786 pregnancies in mid pregnancy at 23.9 (IQR 20.7–26.3) weeks and 979 pregnancies in late pregnancy at 34.1 (IQR 32.1–35.9) weeks.

### Dietary patterns in pregnancies with and without type 1 diabetes

Two principal components together explained 29% of the variation amongst the 19 food item categories, and they were retained to best describe the dietary patterns of all participants (Table 2; ESM Fig. 1). The first component was termed ‘processed food’ because of the high loading for processed snacks, red and processed meat, pizza and refined grains. The second component was termed ‘fresh food’ because of the high loading for nuts, vegetables and fruit.

**Table 2:** Factor loadings of different food item categories in the two dietary patterns during pregnancy identified using PCA in women with and without type 1 diabetes (*n*=983)

Food item category	Loading	
	‘Processed food’ dietary pattern	‘Fresh food’ dietary pattern
Vegetables	0.12	0.41
Red and processed meats	0.34	−0.07
Butter and margarine	0.20	−0.14
Dairy	0.15	0.01
Alcohol	0.03	0.04
Whole grains	0.19	0.26
Sugar	0.11	−0.28
Processed snacks	0.35	−0.02
Refined grains	0.31	−0.06
Poultry	0.28	0.05
Pizza	0.33	−0.16
Nuts	0.11	0.47
Savoury pastries	0.28	−0.20
Fruit juice	0.22	−0.18
Fruit	0.12	0.35
Fish	0.20	0.31
Eggs	0.04	0.27
Condiments	0.29	0.08
Chips/French fries	0.28	−0.19
% of variance	18.02	10.95

Women with type 1 diabetes were more likely to have a ‘fresh food’ pattern (OR 1.19, 95% CI 1.07, 1.31; *p*=0.001) and less likely to have a ‘processed food’ pattern than those without type 1 diabetes (OR 0.89, 95% CI 0.82, 0.96; *p*=0.002). Women with type 1 diabetes who were in the highest quartile of the ‘fresh food’ pattern principal component scores were older, had a lower BMI and HbA_1c_, and a higher socioeconomic status than women with type 1 diabetes in the lowest quartile (Table 3). The median values of servings for most food groups were below the Australian Dietary Guideline recommendations for both groups.

**Table 3:** Maternal, infant and dietary characteristics across quartiles of ‘fresh foods’ dietary pattern score in women with type 1 diabetes (*n*=615)

Characteristic	1st quartile (*n*=154)	2nd quartile (*n*=154)	3rd quartile (*n*=153)	4th quartile (*n*=154)
Age at delivery (years)	30.0 (4.6)	32.0 (4.3)	32.9 (4.7)	32.4 (4.0)
Born in Australia, *n* (%)	134 (87)	121 (79)	128 (84)	119 (77)
Missing	1 (1)	5 (3)	0	2 (1)
IRSAD, *n* (%)				
Quintile 1 (most disadvantaged)	25 (16)	18 (12)	6 (4)	4 (3)
Quintile 2	23 (15)	18 (12)	23 (15)	14 (9)
Quintile 3	39 (25)	27 (18)	27 (18)	24 (16)
Quintile 4	37 (24)	47 (31)	46 (30)	44 (29)
Quintile 5 (least disadvantaged)	30 (19)	44 (29)	51 (33)	68 (44)
Remoteness, *n* (%)				
Metropolitan area	130 (84)	130 (84)	128 (84)	124 (81)
Regional	23 (15)	24 (16)	22 (14)	25 (16)
Remote	1 (1)	0	3 (2)	5 (3)
Education, *n* (%)				
Tertiary	96 (62)	125 (81)	135 (88)	135 (88)
Secondary or less	58 (38)	26 (17)	17 (11)	17 (11)
Missing	0	3 (2)	1 (1)	2 (1)
Parity, *n* (%)				
0	73 (47)	78 (51)	75 (49)	82 (53)
1	59 (38)	52 (34)	55 (36)	56 (36)
2+	22 (14)	24 (16)	23 (15)	16 (10)
Pre-pregnancy BMI (kg/m^2^)	27.6 (23.5–32.2)	25.6 (22.9–30.1)	25.0 (22.5–28.0)	23.8 (22.0–26.1)
Obesity (BMI >30 kg/m^2^), *n* (%)	58 (38)	41 (27)	29 (19)	21 (14)
Missing	1 (1)	1 (1)	1 (1)	1 (1)
Gestational weight gain (kg)	11.4 (7.30–16.0)	12.8 (9.50–17.0)	13.0 (10.0–16.0)	13.1 (11.0–17.0)
Missing, *n* (%)	4 (3)	3 (2)	5 (3)	4 (3)
HbA_1c_ (mmol/mol)	56 (46–66)	52 (46–58)	51 (45–57)	48 (43–54)
HbA_1c_ (%)	7.3 (6.4–8.2)	6.9 (6.4–7.5)	6.8 (6.3–7.4)	6.5 (6.1–7.1)
Missing, *n* (%)	4 (3)	6 (4)	0	2 (1)
Any smoking during pregnancy, *n* (%)	22 (14)	10 (6)	7 (5)	8 (5)
Missing	0	0	2 (1)	3 (2)
HDP, *n* (%)				
Gestational hypertension	18 (12)	13 (8)	13 (8)	13 (8)
Pre-eclampsia	25 (16)	25 (16)	17 (11)	9 (6)
Superimposed pre-eclampsia	5 (3)	5 (3)	3 (2)	2 (1)
Eclampsia	0	0	0	0
HELLP syndrome	1 (1)	1 (1)	2 (1)	2 (1)
Missing	0	0	0	4 (3)
Gestational age at birth (weeks)	37.0 (36.0–37.7)	37.1 (36.1–38.0)	37.3 (36.6–37.9)	37.9 (37.1–38.4)
Premature (<37 weeks), *n* (%)	75 (49)	61 (40)	49 (32)	30 (19)
Term (37–41 weeks), *n* (%)	79 (51)	93 (60)	104 (68)	124 (81)
Birthweight (g)	3632 (3182–3990)	3665 (3295–4050)	3720 (3235–4130)	3686 (3320–4000)
Small for gestational age^a^, *n* (%)	0	0	2 (1)	0
Large for gestational age^b^, *n* (%)	109 (71)	98 (64)	100 (65)	84 (55)
Missing, *n* (%)	0	0	0	1 (1)
Neonatal hypoglycaemia, *n* (%)	116 (75)	117 (76)	104 (68)	98 (64)
Missing	0	0	0	1 (1)
Food groups (number of servings^c^)				
Fruit (2)	1.23 (0.78)	1.75 (1.12)	1.89 (0.84)	2.38 (1.01)
Vegetables (5)	1.43 (0.73)	1.67 (0.71)	2.09 (0.86)	2.55 (0.85)
Dairy (2.5)	1.76 (0.88)	1.95 (0.86)	2.10 (0.91)	2.06 (0.82)
Grains (8.5)	3.18 (2.11)	3.36 (2.11)	3.41 (1.55)	3.56 (2.67)
Meat and alternatives (3.5)	1.71 (1.24)	1.87 (1.00)	2.26 (0.95)	2.85 (1.44)
Discretionary items (0–2.5)	3.43 (1.99)	3.26 (2.05)	3.11 (1.94)	2.68 (1.76)
Alcohol (0)	0.02 (0.14)	0.04 (0.20)	0.04 (0.29)	0.02 (0.23)

Data are presented as mean (SD), median (IQR; quartile 1–quartile 3) or *n* (%)

If missing data are not included, there were no missing values for that variable

^a^Small for gestational age defined as ≤10th centile from population-based Australian birthweight centile charts [43]

^b^Large for gestational age defined as ≥90th centile from population-based Australian birthweight centile charts [43]

^c^Recommended daily number of servings from each food group during pregnancy reported in parentheses [17]

### Association between dietary patterns and maternal complications and birth outcomes in pregnancies with type 1 diabetes

**Fig. 2:**
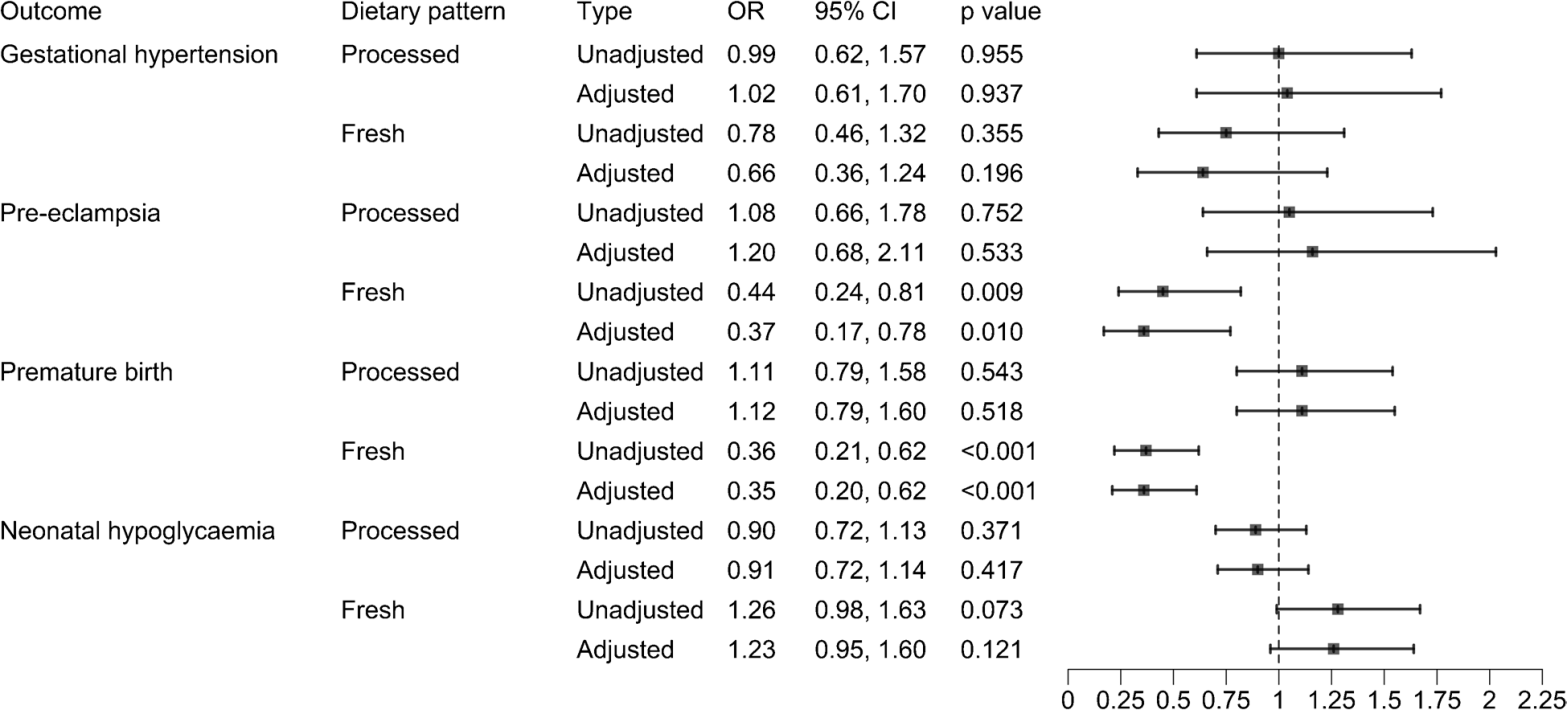
Association between dietary patterns (‘processed food’ dietary pattern or ‘fresh food’ dietary pattern) and maternal complications and birth outcomes in women with type 1 diabetes (*n*=615). Values are presented as ORs with 95% CIs for an increase of one IQR in dietary pattern principal components score. Models were adjusted for age, parity and socioeconomic status

In women with type 1 diabetes, a one-unit increase in the rescaled scores of the ‘fresh food’ pattern was associated with a decreased likelihood of pre-eclampsia and premature birth (Fig. 2). The ‘fresh food’ pattern was associated with an increased gestational age at birth (β coefficient 0.38 weeks, 95% CI 0.23, 0.53; *p*<0.001). No association was detected between dietary pattern and gestational hypertension. The ‘processed food’ pattern was associated with an increased birthweight (β coefficient 56.8 g, 95% CI 2.8, 110.8; *p*=0.04). There were no associations between dietary pattern and neonatal hypoglycaemia. A diet more aligned with the ‘fresh food’ pattern was associated with modestly lower HbA_1c_ (β coefficient −2.79 mmol/mol, 95% CI −4.20, −1.38; *p*<0.001) and a diet more aligned with the ‘processed food’ pattern was associated with a higher HbA_1c_ (β coefficient 1.31 mmol/mol, 95% CI 0.06, 2.55; *p*=0.039).

The difference in food group servings between the highest and lowest quartile of principal component scores for the ‘fresh food’ dietary pattern was, on average, an additional 1.15 servings of fruit, 1.12 servings of vegetables, 0.30 serving of dairy, 0.38 serving of grains, and 1.14 servings of lean meats, poultry, fish, eggs, tofu, legumes/beans; and 0.75 less serving of discretionary items (foods high in calories, saturated fat, added sugar and/or added salt).

### Association between physical activity and maternal complications and birth outcomes in pregnancies with type 1 diabetes

Total physical activity was associated with gestational age at birth (β coefficient 0.06 weeks, 95% CI 0.01, 0.11; *p*=0.014) and birthweight (β coefficient 36.6 g, 95% CI 16.4, 56.7; *p*<0.001) in the unadjusted models only. Sedentary activity was associated with birthweight (β coefficient −58.8 g, 95% CI −79.2, −38.0; *p*<0.001) in the unadjusted model only. Moderate and vigorous activity was associated with birthweight (β coefficient 30.2 g, 95% CI 10.2, 50.3; *p*=0.003) in the unadjusted model only. There were no other associations between physical activity and either maternal complications or birth outcomes in the adjusted models.

### Mediation analyses

Separate mediation analyses showed that 27% (*p*=0.026) of the effect of the ‘fresh food’ pattern on pre-eclampsia and 23% (*p*<0.001) of the effect on premature birth was the result of the mediation pathway through HbA_1c_, while 26% (*p*=0.030) of the effect on pre-eclampsia and 14% (*p*=0.006) of the effect on premature birth was a result of the mediation pathway through maternal BMI (ESM Fig. 2).

### Sensitivity analyses

Removing women with other types of diabetes from the women without type 1 diabetes group when undertaking PCA did not change the associations between dietary patterns and outcomes. After excluding participants who reported an unrealistic energy intake and refitting all models, the standard errors of model coefficients increased slightly for ‘fresh food’ dietary pattern and pre-eclampsia, as expected from a small reduction in the sample size (ESM Table 2a). In addition, an increase in the ‘processed food’ pattern was now associated with an increased likelihood of gestational hypertension (OR 2.29, 95%CI 1.11, 4.72; *p*=0.024) and premature birth (OR 1.54, 95% CI 1.04, 2.27; *p*=0.030), and a decrease in gestational age at birth (β coefficient −0.16 weeks, 95% CI −0.31, −0.01; *p*=0.033). A sensitivity analysis of only nulliparous participants showed no changes for pre-eclampsia and premature birth (ESM Table 2b).

## Discussion

We report the first large comprehensive investigation of dietary patterns and physical activity during pregnancy in 973 women (1124 pregnancies) with and without type 1 diabetes followed prospectively in the same cohort, and their association with complications and birth outcomes in women with type 1 diabetes. In 615 women with type 1 diabetes, a ‘fresh food’ dietary pattern was associated with a reduction in risk of pre-eclampsia by 63% and of premature birth by 65%. Importantly, relatively small increments in fresh food intake in the daily diet separated those women with type 1 diabetes in the highest quartile from those in the lowest quartile of the ‘fresh food’ pattern, suggesting that sustained small changes in daily intake could be associated with sizeable improvements in outcomes. A lower score for the ‘fresh food’ pattern was associated with modestly higher HbA_1c_ levels, where HbA_1c_ was one mediator, along with BMI, of the associations between diet and maternal complications and birth outcomes. Women with type 1 diabetes were more likely to have a ‘fresh food’ dietary pattern in pregnancy than those without type 1 diabetes. Even so, the majority of women did not meet Australian dietary recommendations for pregnancy, irrespective of their type 1 diabetes status.

The approximately fivefold increased risk of both pre-eclampsia and premature birth in women with type 1 diabetes make our findings, and the opportunities they present for dietary intervention, particularly relevant. Dietary patterns characterised by high intake of foods with antioxidative and anti-inflammatory properties such as vegetables, fruits, wholegrains, fish, legumes and pulses, predominant in the ‘fresh food’ pattern, may reduce inflammation that contributes to both pre-eclampsia and premature birth [26]. Many women find it difficult to consume recommended dietary intakes during pregnancy [27–30]. Consistent with other international studies of type 1 diabetes in pregnancy [31], the ENDIA mothers’ mean intakes, even in the highest quartile of the ‘fresh food’ dietary pattern, only met recommended fruit and dairy intakes. To therefore better estimate dietary changes in women with type 1 diabetes that would be needed to reduce risk of pre-eclampsia and premature birth, we looked at the mean differences in food group serving intake between the lowest and highest quartile of principal component scores for the ‘fresh food’ dietary pattern. Reassuringly, this equated to relatively small increments per day: one-third more serving of both dairy and grains, and one serving of lean meat, one serving of fruit, one serving of vegetables and three-quarter less serving of discretionary items or junk food. Dietary patterns with higher intakes of fruits and vegetables have been associated with similar sized risk reduction in pre-eclampsia and premature birth in the general population as we report in type 1 diabetes [3, 5]. Dietary interventions in the general population have also reduced these complications, which is encouraging for women with type 1 diabetes in light of our findings [32, 33].

Limited studies have examined physical activity levels during pregnancy in type 1 diabetes [34, 35]. The lack of association that we describe for adjusted models between exercise and HDP and birth outcomes has been described by others, but for early pregnancy exercise only, and in smaller numbers [35]. Sedentary behaviour in early pregnancy may be higher in women developing pre-eclampsia [35] but larger studies are needed. Despite reporting higher levels of total physical activity than other studies [35, 36], we found no beneficial association with outcomes.

Strengths of our study are the multi-centre design and, to our knowledge, this is the largest single prospective study globally to investigate dietary patterns and physical activity in pregnancies with type 1 diabetes. The ENDIA participants were relatively comparable with the population who gave birth in Australia [37]. They were of similar age, parity and rates of overweight and obesity, although a higher proportion of ENDIA participants were born in Australia, achieved tertiary education and had modestly higher socioeconomic status. Further, rates of pregnancy complications and birth outcomes were comparable with other type 1 diabetes in pregnancy cohorts in Australia [38, 39] and Europe [40, 41].

Our study has several limitations. First, as above, higher socioeconomic status and level of education may limit the generalisability to all pregnancies with type 1 diabetes. As positive social determinants of health, they may influence the diet, physical activity and pregnancy/birth outcomes. Although the food frequency questionnaire is a valid and reliable tool covering long-term food intake, it is self-reported and limited by memory recall and the foods listed in the questionnaire. Participants may have been influenced more by their current dietary intakes in their third trimester. Over- and under-reporting is common with food frequency questionnaires so a sensitivity analysis was conducted excluding those with ‘unrealistic’ energy intakes. A ‘processed food’ dietary pattern was then associated with increased risk of gestational hypertension (2 times the odds) and premature birth (1.5 times the odds) in women with type 1 diabetes; this additional finding is consistent with the main findings of the study. The majority of ‘unrealistic’ reporters under-reported their dietary intake (97%). Foods with a negative health image, like processed foods, are more likely to be under-reported [42]. Serving size for discretionary items may also be smaller than expected (e.g. half a chocolate bar). While HbA_1c_ is a practical measure of glycaemic control, it cannot assess glucose variability. Continuous glucose monitoring would have provided a more comprehensive assessment of glycaemic control and variability, but these metrics were not available. In relation to maternal BMI, pre-pregnancy weights were self-reported as participants enrolled after they became pregnant. Australian maternity practice has changed recently to not weigh women regularly during pregnancy, which also limited the number of data points for gestational weight gain. A further clinical practice is that large for gestational age babies and babies from pregnancies with type 1 diabetes are often treated prophylactically for hypoglycaemia, thus limiting our ability to assess the association of diet and this outcome.

In conclusion, the benefits associated with a diet higher in fresh food in reducing pre-eclampsia and prematurity in the general population are also detectable in type 1 diabetes. Even though most women were not meeting the Australian Dietary Guidelines, it is a reassuring message that smaller, more achievable differences in dietary intake have the potential to reduce the risk of pre-eclampsia and premature birth significantly. This is encouraging for women with type 1 diabetes, who bear the burden of a much higher risk of these complications. Our findings offer the potential to reduce their risk of pre-eclampsia and premature birth with early pre-conception and systematic advice of the benefits of a fresh food diet rich in vegetables, fruit, nuts, fish and grains.

## Supplementary Information

Below is the link to the electronic supplementary material.

ESM (PDF 252 KB)

## Data Availability

Data used in this study were derived from the ENDIA study. Data from the ENDIA study are available upon reasonable request via email to the corresponding author or endia@adelaide.edu.au.

## Abbreviations

DQESv2: Dietary Questionnaire for Epidemiological Studies version 2
ENDIA: Environmental Determinants of Islet Autoimmunity
HDP: Hypertensive disorders of pregnancy
HELLP: Haemolysis, elevated liver enzymes and low platelets
MET: Metabolic equivalent of task
PCA: Principal component analysis
PPAQ: Pregnancy Physical Activity Questionnaire
SEIFA IRSAD: Socio-Economic Indexes for Areas Index of Relative Socio-Economic Advantage and Disadvantage
