# Supplementary material for: Dietary patterns during pregnancy and maternal and birth outcomes in women with type 1 diabetes: the Environmental Determinants of Islet Autoimmunity (ENDIA) study
Source: Diabetologia. 2024 Sep 2;67(11):2420–32. doi: 10.1007/s00125-024-06259-5 (PMC11519125; doi:10.1007/s00125-024-06259-5)
Supplement: Supplementary file 1 — ESM (PDF 252 KB) [file 125_2024_6259_MOESM1_ESM.pdf]

Electronic supplementary material (ESM)

Dietary patterns during pregnancy and maternal and birth outcomes in women with type 1 diabetes: the Environmental Determinants of Islet Autoimmunity (ENDIA) study

Rebecca L. Thomson, James D. Brown, Helena Oakey, Kirsten Palmer, Pat Ashwood, Megan A. S. Penno, Kelly J. McGorm, Rachel Battersby, Peter G. Colman, Maria E. Craig, Elizabeth A. Davis, Tony Huynh, Leonard C. Harrison, Aveni Haynes, Richard O. Sinnott, Peter J. Vuillermin, John M. Wentworth, Georgia Soldatos, Jennifer J. Couper, on behalf of the ENDIA Study Group

Page	Item
**2**	ESM Table 1. Food items used in the dietary pattern analysis
**3**	ESM Fig. 1. Scree plot for identification of dietary patterns by principal components analysis
**4**	ESM Fig. 2. Mediation analysis
**5**	ESM Table 2A. Sensitivity Analysis Results - Model parameters from sensitivity analysis model 1
**6**	ESM Table 2B. Sensitivity Analysis Results - Model parameters from sensitivity analysis model 2
**7-8**	ESM File 1. Composition of ENDIA Study Group

ESM Table 1. Food items used in the dietary pattern analysis

Food Category	Food items*
Red and processed meats	Beef, pork, lamb, veal, *hamburger, bacon, ham, salami, corned beef, luncheon meats, sausages*
Fish	Fish (steamed, grilled or baked), *fried fish*, tinned fish (salmon, tuna, sardines)
Poultry	Chicken
Eggs	Eggs
Butter and margarine	*Butter*, margarine, poly/mono-unsaturated margarine, butter and margarine blends
Dairy	Reduced fat, skim and full cream milk, low fat, hard, firm, soft, ricotta, cottage or cream cheese, yogurt, *ice cream*, flavoured milk drink
Alcohol	Red, white and fortified wine, port, sherry, light and heavy beer, spirits and liqueurs
Fruit	Tinned or frozen fruit, orange or other citrus fruit, apple, pear, banana, watermelon, rockmelon, honeydew, pineapple, strawberry, apricot, peach, nectarine, mango or paw paw
Fruit juice	Fruit juice
Vegetables	Cabbage or Brussels sprouts, cauliflower, broccoli, carrot pumpkin, fresh or tinned tomatoes, tomato sauce, tomato paste, dried tomato, lettuce, endive or other salad greens, spinach or silver beet, peas, green beans, bean or alfalfa sprouts, baked beans, tofu, soy beans, soy bean curd, other beans (chickpeas, lentils, etc.), soya milk, cucumber, celery, beetroot, mushroom, zucchini, capsicum, avocado, onion, leek, garlic, potatoes cooked without fat
Chips/French fries	*Roasted or fried potatoes, including hot chips*
Whole grains	All-Bran, Sultana Bran, FibrePlus, bran flakes, wheat biscuits, cornflakes, Nutrigrain, Special K, porridge, muesli, wholemeal/rye/multi-grain bread
Refined grains	High-fibre white/white bread, rice, pasta, noodles, crackers, crispbreads, dry biscuits
Pizza	*Pizza*
Processed snacks	*Sweet biscuits, cakes, sweet pies, tarts and other sweet pastries, potato crisps, corn chips, Twisties, chocolate*
Nuts	Nuts, peanut butter or paste
Condiments	*Jam, marmalade, honey, syrups*, Vegemite, Marmite, Promite
Sugar	Sugar
Savoury pastries	*Meat pies, pasties, quiche and other savoury pastries*

**Italic food items were identified as discretionary foods*

Adapted from Ashby-Mitchell K, Peeters A, Anstey KJ (2015) Role of dietary pattern analysis in determining cognitive status in elderly Australian adults. Nutrients 7(2): 1052-1067.

ESM Fig. 1. Scree plot for identification of dietary patterns (components) by principal components analysis

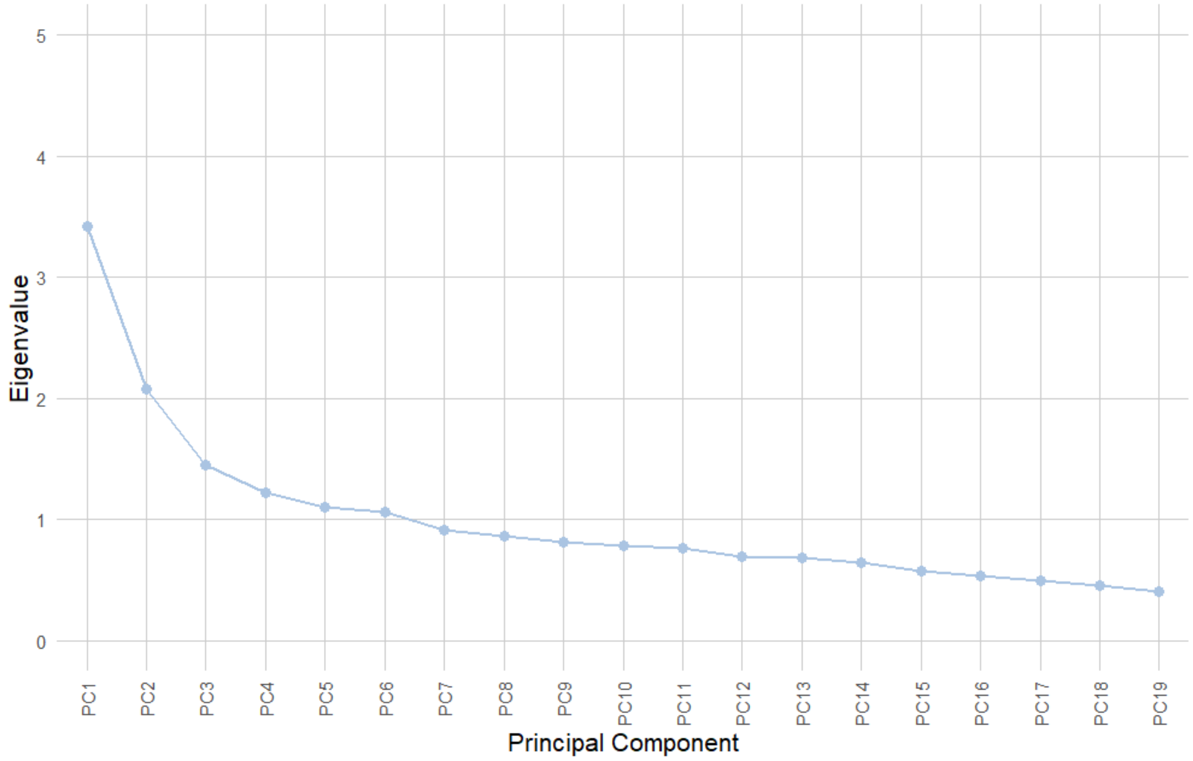

The number of dietary patterns (principal components) identified was based on eigenvalues >1.5 and on identification of a break point in the scree plot. Two principal components together explained 29% of the variation amongst the 19 food item categories, and they were retained to best describe the dietary patterns of all participants.

ESM Fig. 2. Mediation analysis

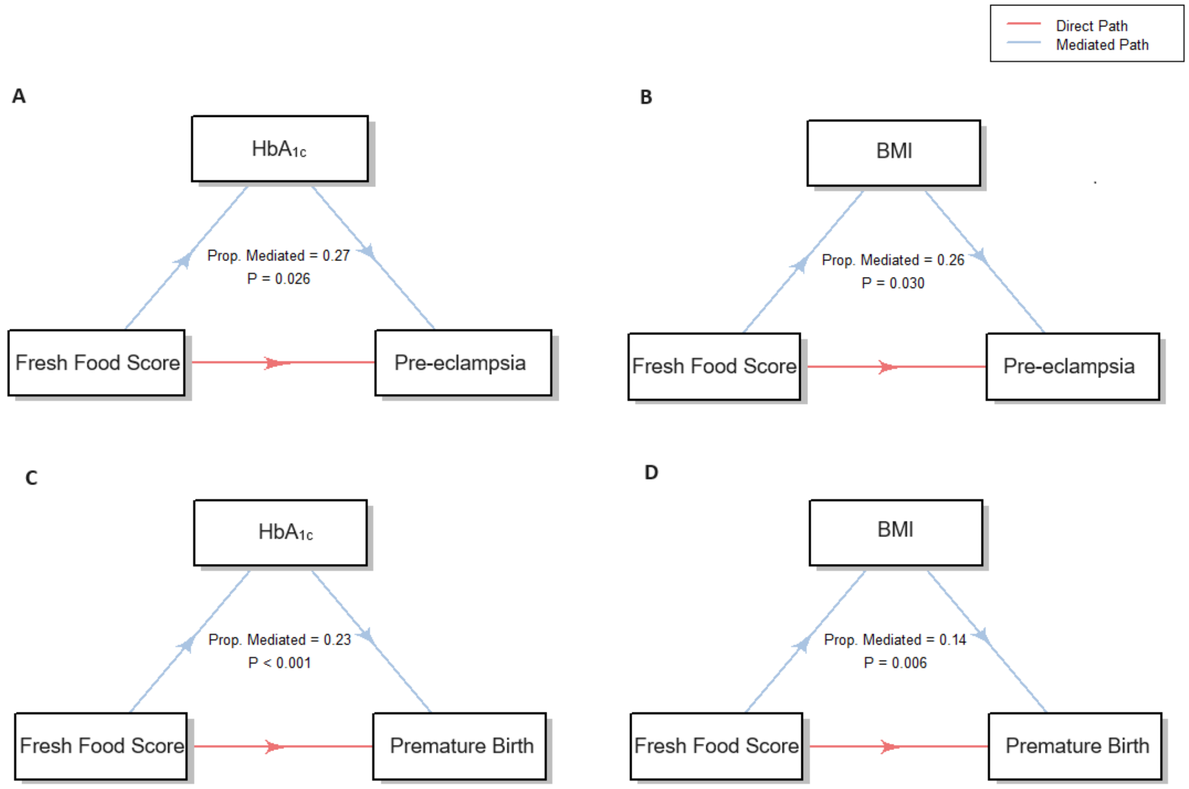

Mediation models assessing the association between the 'fresh food' dietary pattern score and preeclampsia and premature birth. A) mediation analysis showing that 27% (p=0.026) of the effect of the 'fresh food' pattern on preeclampsia was the result of the mediation pathway through HbA1c and B) 26% (p=0.030) of the effect on preeclampsia was a result of the mediation pathway through maternal BMI. C) mediation analysis showing that 23% (p<0.001) of the effect of the 'fresh food' pattern on premature birth was the result of the mediation pathway through HbA1c and D) 14% (p=0.006) of the effect on premature birth was a result of the mediation pathway through maternal BMI.

Model-based causal mediation analysis with quasi-Bayesian Monte Carlo simulation (10,000 simulation) was performed using the 'mediation' R package.

ESM Table 2. Sensitivity Analysis Results

A: Model parameters from sensitivity analysis model 1

This analysis includes 533 participants with type 1 diabetes. Two participants with type 1 diabetes who consumed more than 20,000 kJ per day were removed and 80 participants with type 1 diabetes who consumed less than 4500 kJ were removed. Participants diagnosed with type 2 diabetes (n=1) and gestational diabetes (n=51) were also excluded from the principal component analysis (although they were not included in the models as this was only in participants with type 1 diabetes; n=824 participants with and without type 1 diabetes included in the principal component analysis). For all birth and maternal outcomes, a random intercept for gestational mother was included to account for the correlation between the data from the same mother at different pregnancies.

Outcome	Dietary pattern	Odds ratio[a]/β coefficient[b] (95% confidence interval)			
		Unadjusted	*p* value	Adjusted[c]	*p* value
Pre-eclampsia[a]	Processed	1.20 (0.66, 2.19)	0.551	1.56 (0.69, 3.55)	0.286
	Fresh	0.43 (0.21, 0.88)	**0.022**	0.33 (0.10, 1.06)	0.063
Gestational hypertension[a]	Processed	1.93 (1.02, 3.63)	**0.042**	2.29 (1.11, 4.72)	**0.024**
	Fresh	1.00 (0.55, 1.81)	0.996	0.94 (0.49, 1.81)	0.857
Premature birth[a]	Processed	1.53 (1.05, 2.22)	**0.027**	1.54 (1.04, 2.27)	**0.030**
	Fresh	0.48 (0.30, 0.76)	**0.002**	0.47 (0.29, 0.76)	**0.002**
Gestational age at birth[b]	Processed	-0.18 (-0.33, -0.03)	**0.016**	-0.16 (-0.31, -0.01)	**0.033**
	Fresh	0.34 (0.18, 0.50)	**<0.001**	0.33 (0.16, 0.49)	**<0.001**
Birthweight[b,d]	Processed	49.8 (-15.5, 115.1)	0.135	60.8 (0.2, 121.3)	**0.049**
	Fresh	38.4 (-32.0, 108.7)	0.284	-12.1 (-78.7, 54.6)	0.722
Neonatal hypoglycaemia[a]	Processed	0.85 (0.67, 1.10)	0.218	0.87 (0.68, 1.12)	0.291
	Fresh	1.11 (0.85, 1.45)	0.441	1.09 (0.83, 1.44)	0.526
Maternal HbA_{1c}[b]	Processed	2.56 (1.14, 3.97)	**<0.001**	1.58 (0.18, 2.97)	**0.027**
	Fresh	-5.14 (-6.68, -3.60)	**<0.001**	-3.65 (-5.21, -2.08)	**<0.001**

Fresh = fresh food dietary pattern; Processed = processed food dietary pattern

[a]odds ratio reported from mixed logistic regression models to estimate associations between dietary pattern (principal component scores) and each outcome. In order to make the results more interpretable clinically, the principal components scores were rescaled such that a one-unit change represents the difference from the end of quartile 1 (25th percentile) to the end of quartile 3 (75th percentile).

[b]β coefficient reported from linear mixed models used to estimate associations between diet and each outcome.

[c]models were adjusted for maternal age at birth, parity and socioeconomic status; [d]birthweight was also adjusted for gestational age at birth.

B: Model parameters from sensitivity analysis model 2

This analysis includes 308 participants with type 1 diabetes with a parity of 0. Participants with a parity greater than 0 were excluded (n=307). For all birth and maternal outcomes, a random intercept for gestational mother was included to account for the correlation between the data from the same mother at different pregnancies.

Outcome	Dietary pattern	Odds ratio[a] (95% confidence interval)			
		Unadjusted	*p* value	Adjusted[b]	*p* value
Pre-eclampsia	Processed	1.03 (0.71, 1.50)	0.882	1.04 (0.71, 1.52)	0.846
	Fresh	0.66 (0.45, 0.96)	**0.033**	0.67 (0.44, 1.00)	0.052
Gestational hypertension	Processed	1.25 (0.78, 2.03)	0.366	1.25 (0.78, 2.04)	0.355
	Fresh	0.73 (0.45, 1.16)	0.188	0.72 (0.43, 1.18)	0.195
Premature birth	Processed	1.02 (0.74, 1.41)	0.896	1.02 (0.74, 1.41)	0.896
	Fresh	0.54 (0.39, 0.75)	**<0.001**	0.54 (0.39, 0.75)	**<0.001**

Fresh = fresh food dietary pattern; Processed = processed food dietary pattern

[a]odds ratio reported from mixed logistic regression models to estimate associations between dietary pattern (principal component scores) and each outcome. In order to make the results more interpretable clinically, the principal components scores were rescaled such that a one-unit change represents the difference from the end of quartile 1 (25th percentile) to the end of quartile 3 (75th percentile).

[b]models were adjusted for maternal age at birth, parity and socioeconomic status.

ESM File 1. Composition of ENDIA Study Group

The ENDIA Study Group (March 2024) is composed of: Simon C. Barry (Robinson Research Institute, University of Adelaide, Australia), Emma Hamilton-Williams (Frazer Institute, The University of Queensland, Australia), Ki Wook Kim (Discipline of Paediatrics and Child Health, University of New South Wales, Australia), Grant Morahan (Harry Perkins Institute of Medical Research, The University of Western Australia, Australia), William D. Rawlinson (Serology and Virology Division, Prince of Wales Hospital, Australia), and Jason Tye-Din (Walter and Eliza Hall Institute, Australia). These individuals are members of the ENDIA Steering Committee.

The ENDIA Study would like to thank the following institutions and individuals for their contribution to ENDIA recruitment and follow-up: Lead Clinical Recruitment/Follow-up Sites: The Women's and Children's Hospital, SA (Jennifer Couper). Royal Melbourne Hospital, Vic (Peter Colman, John Wentworth, Leonard Harrison). Barwon Health, Vic (Peter Vuillermin). Monash Health, Vic (Georgia Soldatos). Children's Hospital at Westmead, NSW (Maria Craig). Royal Hospital for Women, NSW (Maria Craig). St George Hospital, NSW (Maria Craig). John Hunter Children's Hospital, NSW (Prudence Lopez). Princess Margaret Hospital/Perth Children's Hospital, WA (Elizabeth Davis, Aveni Haynes). Mater Mother's Hospital/Queensland Children's Hospital, QLD (Tony Huynh, Mark Harris, Andrew Cotterill). Lead Academic Sites: The University of Adelaide/Robinson Research Institute, SA (Jennifer Couper, Megan Penno, Rebecca Thomson, Kelly McGorm, Helena Oakey, Simon Barry). WEHI, Vic (Leonard Harrison, John Wentworth). University of New South Wales, NSW (Maria Craig, William Rawlinson). University of Sydney, NSW (Maria Craig). University of Western Australia/Telethon Kids Institute/Harry Perkins Institute, WA (Elizabeth Davis, Aveni Haynes, Grant Morahan), University of Melbourne, Vic (Richard Sinnott). University of Queensland, QLD (Mark Harris). Satellite Recruitment/Birthing Sites (SA): Country Health SA (Jennifer Couper), Flinders Medical Centre (Brian Coppin), Lyell McEwin Hospital (Jennifer Couper), Ashford Hospital, Flinders Private Hospital, North Eastern Community Hospital. Satellite Recruitment/Birthing Sites (VIC): Royal Women's Hospital (Alison Nankervis), Ballarat Base Hospital (David Song), Bendigo Health (Mark Savage), Epworth Geelong Hospital (Peter Vuillermin), Mercy Hospital for Women (Christine Houlihan, Peter Colman), St. John of God Geelong (Peter Vuillermin), Sunshine Hospital/Joan Kirner Women's and Children's Hospital (Balasubramanian Krishnamurthy), Werribee Mercy Hospital (Sheetal Tipnis). Satellite Recruitment/Birthing Sites (NSW): Hunter Diabetes Centre (Claire Morbey), John Hunter Hospital (Maria Craig), John Hunter Children's Hospital (Maria Craig, Prudence Lopez), Sydney Children's Hospital (Maria Craig), The Sutherland Hospital (Maria Craig), Westmead Hospital (Maria Craig), North Shore Private Hospital (Maria Craig). Satellite Recruitment/Birthing Sites (WA, all under Elizabeth Davis): Armidale Hospital, Bentley Hospital, Joondalup Health Campus, King Edward Memorial Hospital, Rockingham General Hospital, St John of God Mt. Lawley, St John of God Murdoch, St John of God Subiaco. Satellite Recruitment/Birthing Sites (QLD): Royal Brisbane and Women's Hospital (Michael d'Emden), Wesley Hospital (Stephen Cook, Andrew Cotterill). Satellite Recruitment/Birthing Sites (NT): Royal Darwin Hospital (Louise Maple-Brown). Referring Physicians in Private: Natalie Harrison/Geelong Diabetes & Endocrinology (Vic), Dorothy Graham (WA), Linda McKendrick (SA), Amanda Terry (SA). Other Birthing Hospitals: Albury-Wodonga Hospital, Angliss Hospital, Auburn Public Hospital,

Bankstown Hospital, Bathurst Base Hospital, Beaudesert Hospital, Beijing United Family Hospital (China), Belmont Hospital, Berri Hospital, Blacktown Hospital, Box Hill Hospital, Broome Hospital, Buderim Private Hospital, Bunbury Hospital, Burnside War Memorial Hospital, Caboolture Hospital, Cabrini Hospital Malvern, Cairns Hospital, Cairns Private Hospital, Calvary Hospital, Calvary Hospital Bruce, Calvary Hospital Lenah Valley, Calvary Hospital Wagga, Calvary John James Hospital, Campbelltown Hospital, Canberra Hospital, Casey Hospital, Centenary Hospital for Women and Children, Coffs Harbour Base Hospital, Dandenong Hospital, Darwin Birth Centre, Darwin Private Hospital, Dubbo Base Hospital, Echuca Regional Health, Emerald Hospital, Epworth Freemasons Hospital, Fiona Stanley Hospital, Frances Perry House, Frankston Hospital, Gawler Hospital, Gippsland Health, Glengarry Private Hospital, Gold Coast Private Hospital, Gold Coast University Hospital, Gosford Hospital, Gosford Private Hospital, Goulburn Valley Health, Grafton Base Hospital, Greenslopes Private Hospital, Griffith Base Hospital, Gunnedah Hospital, Hawkes Bay Hospital (New Zealand), Hervey Bay Hospital, Hobart Private Hospital, Hornsby Hospital, Hurstville Private Hospital, Ipswich Hospital, Jessie McPherson Private Hospital, John Flynn Private Hospital, Kalgoorlie Hospital, Kapunda Hospital, Kareena Private Hospital, Katoomba Hospital, Launceston General Hospital, Lismore Base Hospital, Liverpool Hospital, Logan Hospital, Mackay Base Hospital, Maitland Hospital, Manly Hospital, Mater Hospital Sydney, Mater Mothers' Hospital, Mater Mothers' Private Hospital, Mater Private Hospital Mackay, Mater Private Hospital Redland, Mater Women's and Children's Hospital Townsville, Mitcham Private Hospital, Moruya District Hospital, Mount Barker Hospital, Narrabri Hospital, Nepean Hospital, Nepean Private Hospital, Newcastle Private Hospital, North Gosford Private Hospital, North West Hospital, North West Private Hospital, Northeast Health Wangaratta, Northern Beaches Hospital, Northern Hospital, Northpark Private Hospital, Norwest Private Hospital, Orange Base Hospital, Osborne Park Hospital, Peel Health Campus, Peninsula Private Hospital, Pindara Private Hospital, Port Augusta Hospital, Port Macquarie Hospital, Prince of Wales Hospital, Prince of Wales Private Hospital, Queanbeyan District Hospital, Queen Victoria Hospital, Redcliffe Hospital, Redland Hospital, Riverland General Hospital, Rockhampton Base Hospital, Royal Hobart Hospital, Royal Hospital for Women, Royal North Shore Hospital, Royal Prince Alfred Hospital, Sandringham Hospital, St George Private Hospital, St John of God Ballarat Hospital, St John of God Bendigo Hospital, St John of God Berwick Hospital, St John of God Bunbury Hospital, St John of God Midland Hospital, St Vincent's Hospital, St Vincent's Private Hospital, St Vincent’s Private Hospital Toowoomba, Stanthorpe Hospital, Sunnybank Private Hospital, Sunshine Coast University Hospital, Swan District Hospital, Sydney Adventist Hospital, Tamworth Hospital, Tanunda Hospital, The Canberra Hospital, The Tweed Hospital, Toowoomba Base Hospital, Toowoomba Private Hospital, Townsville University Hospital, Wallaroo Hospital, Wangaratta Hospital, Waverley Private Hospital, Westmead Private Hospital, Wodonga Hospital, Wollongong Hospital, Wollongong Private Hospital.
